# Supplementary material for: The causal role of circulating inflammatory markers in osteoporosis: a bidirectional Mendelian randomized study
Source: Front Immunol. 2024 Jul 18;15:1412298. doi: 10.3389/fimmu.2024.1412298 (PMC11291241; doi:10.3389/fimmu.2024.1412298)
Supplement: Supplementary file 1 [file DataSheet_1.zip › supplementary material/OSTEOPOROSIS-CIM/GCST90274772.tsv.gz_leave_one_out.pdf]

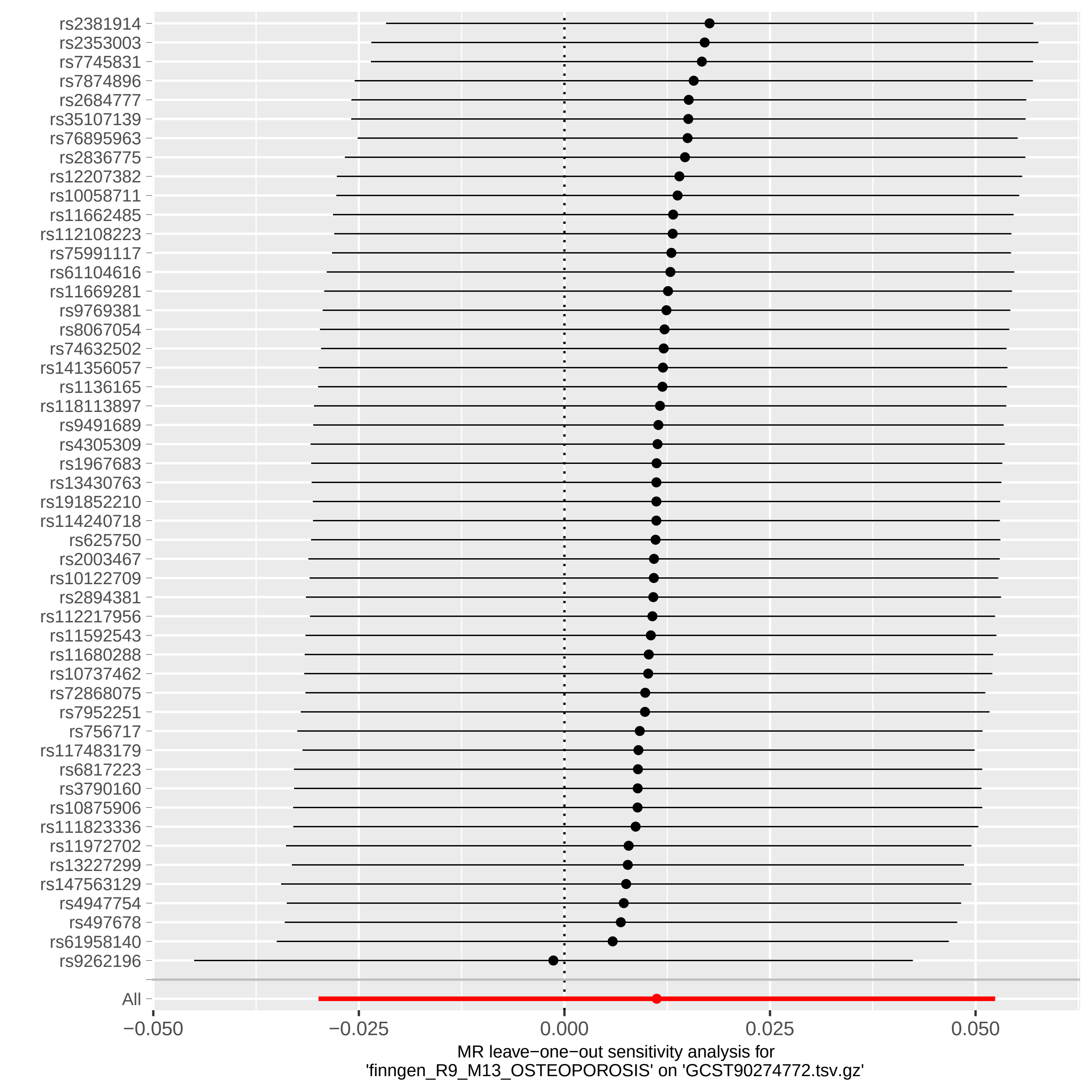

rs2381914
rs2353003
rs7745831
rs7874896
rs2684777
rs35107139
rs76895963
rs2836775
rs12207382
rs10058711
rs11662485
rs112108223
rs75991117
rs61104616
rs11669281
rs9769381
rs8067054
rs74632502
rs141356057
rs1136165
rs118113897
rs9491689
rs4305309
rs1967683
rs13430763
rs191852210
rs114240718
rs625750
rs2003467
rs10122709
rs2894381
rs112217956
rs11592543
rs11680288
rs10737462
rs72868075
rs7952251
rs756717
rs117483179
rs6817223
rs3790160
rs10875906
rs111823336
rs11972702
rs13227299
rs147563129
rs4947754
rs497678
rs61958140
rs9262196
All
−0.050
−0.025
0.000
0.025
0.050
MR leave−one−out sensitivity analysis for
'finngen_R9_M13_OSTEOPOROSIS' on 'GCST90274772.tsv.gz'
